# Recent progress in the effect of magnetic iron oxide nanoparticles on cells and extracellular vesicles

**DOI:** 10.1038/s41420-023-01490-2

**Published:** 2023-06-28

**Authors:** Yuling Chen, Shike Hou

**Affiliations:** 1 Institute of Disaster and Emergency Medicine, Tianjin University, 300072 Tianjin, China; 2 Key Laboratory for Disaster Medicine Technology, 300072 Tianjin, China

**Keywords:** Apoptosis, Mesenchymal stem cells, Stem cells, Mechanisms of disease

## Abstract

At present, iron oxide nanoparticles (IONPs) are widely used in the biomedical field. They have unique advantages in targeted drug delivery, imaging and disease treatment. However, there are many things to pay attention to. In this paper, we reviewed the fate of IONPs in different cells and the influence on the production, separation, delivery and treatment of extracellular vesicles. It aims to provide cutting-edge knowledge related to iron oxide nanoparticles. Only by ensuring the safety and effectiveness of IONPs can their application in biomedical research and clinic be further improved.

## Facts

- What is the fate of IONPs after they enter the cell.
- What is the effect of IONPs after they enter the extracellular vesicles.
- Improve the security and effectiveness of IONPs.

## Introduction

Iron oxide nanoparticles (IONPs) are approved by FDA and are widely used in biomedical research and clinical applications, including magnetic resonance imaging, drug delivery and disease treatment [1–7]. γ-Fe_2_O_3_ and Fe_3_O_4_ nanoparticles have the advantages of biocompatibility and biodegradability [8]. They can be used to label cells and extracellular vesicles (EVs) and track and visualize them through magnetic resonance imaging (MRI) to ensure that they can reach the target site in the body and remain in the tissue, while monitoring the migration and differentiation process of stem cells in the body [9, 10]. However, the level of cytotoxicity induced by IONPs depends on the size, concentration, surface charge, coating type and functional groups of IONPs [11–13]. The uptake of IONPs by cells has an impact on cells and EVs. These effects are both positive and negative. We need to be more vigilant in identifying and responding to risks so that we can make full use of these benefits. Therefore, it is necessary to review and summarize the existing research in order to make better use of the magnetic EVs delivery system.

In the first part of this review, we describe the fate of IONPs after endocytosis in cells. The effects of IONPs on stem cells, macrophages and tumor cells were introduced in detail. The second part focuses on the key role of IONPs in the generation, separation and treatment of EVs, which can stimulate or enhance the secretion of cells to EV. The role of IONPs in the treatment mechanism of EV is of particular interest in nanomedicine. This review will help deepen the understanding of how IONPs affect cells and EVs, enable readers to fully understand the application of magnetic nanomaterials in nanomedicine research, and further promote the development of natural therapeutic and diagnostic nanoplatforms.

## Fate of IONPs in cells

**Fig. 1:**
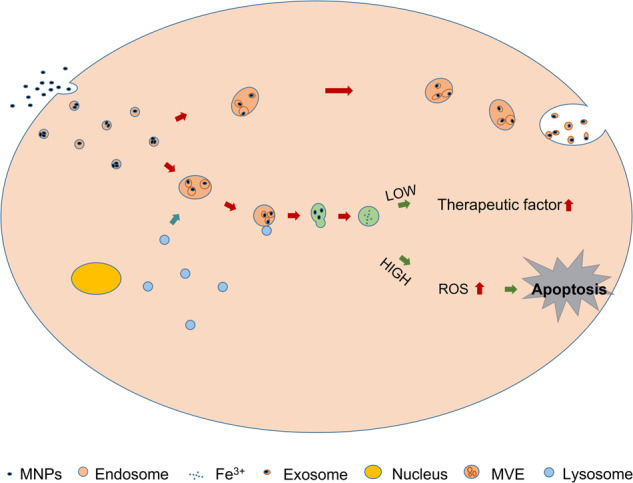
Fate of MNPs in cells.

In vitro culture system, IONPs will be ingested by cells when incubated with cells, as shown in Fig. 1. At the same time, the application of IONPs in the complex blood flow system will also result in the initial uptake of the liver and spleen. In addition, regarding IONPs uptake by cells, researchers found that IONPs uptake was mediated by endocytosis, similar to giant cell drinking [14], after conducting subcellular analysis. The uptake of smaller IONPs is mainly mediated by clathrin- [15] and fossa-mediated endocytosis, while the uptake of larger IONPs and aggregates is mainly mediated by macrocytosis and phagocytosis [12]. In addition, the coating properties of IONPs and cell types determine the internalization mechanism of IONPs [16, 17]. After absorption, IONPs enter the inner lysosome pathway through large cytoplasmic organelles and reach the final destination of multi-layer lysosomes. The confocal microscope showed that the particles were almost completely located in the lysosome compartment after 24 h of co-culture [18]. Exposure to IONPs increases the number, permeability and alkalinity of lysosomes [19]. At the same time, the lysosomal metabolism of superparamagnetic iron oxide nanoparticles (SPION) in cells has been confirmed [20, 21]. SPIONs can be degraded by enzymes in lysosome (pH~4.5) [22, 23], thus releasing free iron in IONPs and affecting iron homeostasis. At the same time, free iron is stored in the form of protein, such as ferritin and hemosiderin, for further use by the host. The biocompatibility of IONPs is attributed to effective cellular iron metabolism, allowing iron circulation and excessive iron exocytosis [24, 25]. In addition, the cytotoxicity of SPIONs to cells largely depends on their size [26, 27], shape, surface coating [17, 28, 29], dose of exposed cells [30], exposure time [31] and type [32–34]. When the exposure concentration of nano-magnetite particles is greater than 50 µg/ml, it will produce greater cytotoxicity in the form of reduced activity, and increase ROS, reduce cell membrane potential and reduce the rate of cell apoptosis [30]. Therefore, as long as the specific parameters of IONPs are controlled, they will not have adverse effects on cell viability, metabolic activity, oxidative stress, proliferation and differentiation [9, 35, 36].

However, iron overload can trigger the generation of reactive oxygen species (ROS) [32, 37]. The main reason for the cytotoxicity of IONPs is the excessive production of ROS. ROS can cause oxidative stress in cells by activating pro-inflammatory mediators, and eventually lead to cell necrosis or apoptosis [38]. Therefore, autophagy induced by IONPs can affect cell metabolism, cytotoxicity, therapeutic effect [39], and even host immune system [40–42].

### Fate of IONPs in stem cells

The therapy based on mesenchymal stem cells (MSCs) has become a promising therapeutic strategy for tissue regeneration and repair. SPION nanoparticles can help stem cells transplant and regenerate in vivo. However, the effect of SPION labeling on the proliferation and differentiation of stem cells is still uncertain. SPION concentration for stem cell labeling is not high (<50 μgFe/ml), does not cause cytotoxicity. For example, the optimal MRI cell tracking dose (about 10 pg Fe/cell) [43]. However, SPION markers have more or less affected several cellular events of mesenchymal stem cells [44–46]. Researchers have found that SPION not only marks cells, but also regulates the differentiation, activity and treatment of stem cells.

### Migrate

After uptake by cells, IONPs are degraded by the nuclear body to release iron ions and further regulate the molecular expression in MSC. CXCR4 and SDF-1 are important factors to induce the homing of MSCs. In order to improve the effectiveness of MSC homing, the internalized SPION in MSC induces the new biological functional characteristics of MSC homing by adding CXCR4 [47–50]. Huang et al. [49] increased CXCR4 expression in MSC and enhanced CXCR4-SDF-1α axis through Zn_0.4_Fe_2.6_O_4_, it is the first time to prove that iron-based can actively increase the expression of chemokine receptor CXCR4 in bone marrow derived MSCs without gene modification, and improve the homing of MSCs to injury sites and tumors. Li et al. [48] also found that Fe_3_O_4_@PDA The incorporation of NPs into MSCs up-regulates the expression level of C-X-C chemokine receptor type 4 (CXCR4) to enhance the migration of MSCs to the site of laser burn injury. In addition, the labeled MSC group showed increased cytokines and decreased pro-inflammatory factors. They also increased the expression of healing-related genes. It is known that SPIO can induce reactive oxygen species (ROS), while ROS can increase CXCR4 expression in bone marrow derived MSCs [51]. The magnetic retention of SPIO-labeled MSCs increases the migration and homing efficiency of MSCs with SPIO nanoparticles in olfactory bulb injured mice [50]. Schulze et al. [52] found that A-PVA-SPIONS accumulated in bone marrow and increased the metabolic activity and migration rate of BMSCs. After Frank et al. [53] labeled with A-PVA-SPION, the migration of MSCs increased.

### Differentiation

The bovine serum albumin (Fe_3_O_4_/BSA) particles of iron oxide nanoparticles significantly promoted the bone formation of mesenchymal stem cells under the action of external magnetic field, while the magnetic particles and external magnetic field alone did not affect the differentiation of stem cells [54]. With the increase of SPION incorporation, cartilage formation, differentiation and chemotaxis are significantly impaired [55]. The damage of cartilage formation may be caused by high intracellular iron load [44, 56]. Ferucarbotran has no toxicity to human mesenchymal stem cells (hMSCs). However, Ferucarbotran causes dose-dependent inhibition of osteogenic differentiation of human mesenchymal stem cells, cancels osteogenic differentiation at high concentrations, promotes cell migration, and activates signal molecules β-Catenin, cancer/testicular antigen, SSX and matrix metalloproteinase 2 (MMP2) [21]. It can also stimulate the proliferation of human mesenchymal stem cells (hMSCs) in the form of particles and ions (free iron, Fe) by reducing intracellular H_2_O_2_, and can affect the expression of cyclin regulators [46].

### Therapy

Connexin 43 (Cx43) induces intercellular coupling between adjacent cells. The formation of gap junction channels containing Cx43 triggered by ionizing IONPs is enhanced. In the treatment of myocardial infarction, IONPs significantly increased the expression of Cx43 in cardiomyocytes (H9C2) [57]. At the same time, IONPs can enhance the selective transfer of intercellular mitochondria from human mesenchymal stem cells (hMSCs) to diseased cells, significantly reducing the process of fibrosis [58]. In addition, SPIONS induce MSCs to secrete angiogenesis, anti-apoptosis and anti-inflammatory factors, such as angiopoietin-1 (Ang-1) and transforming growth factor-β1 (TGF-β1) And vascular endothelial growth factor (VEGF) [47, 48, 59–61]. Therefore, this strengthens the regeneration and repair ability of MSCs.

### Fate of IONPs in macrophage

There is still limited research on the effects of magnetic iron oxide nanoparticles on immune cells. Current studies have found that IOPs activate many biological processes of macrophages, including many immune reactions, endoplasmic reticulum (ER) stress and oxidative stress. SPION processes macrophages to produce reactive oxygen species [62], activates extracellular signal-regulated kinase and AKT pathway [63], JNK signal pathway [18], and promotes the production of IL-10. In addition, IL-1β of bone marrow-derived macrophages (BMMs) in mice was induced in a size-dependent and dose-dependent manner Release [27]. IONPs also induce BMMs to produce IL-6 and tumor necrosis factor α(TNF-α) [64]. Iron in SPION induces THP1-derived M2 macrophages to high CD86^+^and high TNF α^+^ Macrophage subtype transformation [65]. Fe_3_O_4_ nanoparticles can reduce the activity of macrophages and induce the polarization of macrophages to M1 phenotype after 48 h of treatment [66]. In addition, Fe_3_O_4_ nanoparticles induce iron death in macrophages. P53 may contribute to iron death of macrophages induced by Fe_3_O_4_ nanoparticles. It provides a theoretical basis for the molecular mechanism of iron death of macrophages and Fe-induced biological toxicity in vivo.

### The fate of IONPs in cancer cells

Iron death is an iron-dependent cell death caused by excessive oxidation of polyunsaturated fatty acids. It can be activated by iron-based nanoparticles for cancer treatment. Fe_3_O_4_ magnetic nanoparticles are more sensitive to weak acids. When entering cancer cells, it can release abundant Fe (II) ions to participate in Fenton reaction in combination with acidic tumor microenvironment and internal body conditions, and then catalyze H_2_O_2_ to become highly toxic OH•, which will increase cell oxidative stress, damage mitochondria and cell membrane and induce tumor cell iron death [67], induce autophagy [68, 69], apoptosis and necrosis [70]. However, it is usually difficult to produce enough •OH, which is limited by intracellular H_2_O_2_ concentration to induce iron death of cancer cells [71]. In order to achieve sufficient anti-tumor effect. It is reported that the intracellular cascade reaction can increase the concentration of H_2_O_2_ in cells, thus improving the high-level production of •OH through the joint transport of metal catalysts and H_2_O_2_ producers [72, 73]. Cisplatin can indirectly produce H_2_O_2_ to further accelerate Fenton reaction. It can induce intracellular cascade reaction to produce enough •OH for iron death treatment [74]. In addition, the liposome is embedded with PEG-coated The liposome bilayer of γ-Fe_2_O_3_ nanoparticles improves the permeability of H_2_O_2_ and •OH [75], resulting in the effective activation of lipid peroxidation [76]. Moreover, 808 nm laser irradiation heat-induced Fe_3_O_4_ in situ burst release produces effective reactive oxygen species through Fenton reaction in tumor microenvironment [77].

The cytotoxicity of superparamagnetic particles depends on the size, concentration, particle shape and coating type [78]. With the increase of particle concentration within the range of cytotoxicity [79]. With the increase of the number of IONPs, the released iron level will increase, which will catalyze the formation of oxidative free radicals, leading to cell death. Before reaching the high exposure level, although there is no obvious cytotoxicity, it has an impact on cells. For example, Watanabe et al. [80] found that lower concentrations would increase the production of reactive oxygen species, increase oxidative damage to DNA [22], and reduce the level of reduced glutathione. MgNPs-Fe_3_O_4_ only at high concentration (100 μg/mL), the release of lactate dehydrogenase (LDH) was used to evaluate the cell membrane damage [80]. In addition, with the deepening of research, Fe ions generate a lot of toxic ROS. It also promoted the down-regulation of GPX4 [81] and xCT [82], and lipid peroxide began to accumulate. Apoptosis of caspase-8 activated cells after short time exposure to AC magnetic field [36]. Zhou et al. [83] showed that gold-plated iron oxide particles(Fe_3_O_4_@Au SPs) can induce non-cytotoxicity and gene expression in tumor cells, which are mainly involved in cell proliferation, differentiation and apoptosis. At clinically relevant doses, ferumoxytol has no direct cytotoxic effect on cancer cells. Adenocarcinoma cells incubated with ferumoxytol and macrophages showed increased caspase-3 activity. Zanganeh et al. [39] found that ferumoxytol caused tumor growth inhibition through indirect influence on tumor microenvironment.

At present, the joint action of SPION and external magnetic field has a far-reaching impact in many fields of nanotechnology. It can not only achieve long-distance and accurate targeted delivery, but also transform energy into force or heat for disinfection and sterilization tumor cells under the action of alternating magnetic field [84, 85]. Because SPION retained in lysosomal compartment can cause lysosomal membrane permeability (LMP) in cancer cells to cause mechanical destruction of lysosomes through high intensity dynamic magnetic field [86]. The contents of apoptotic lysozyme infiltrated into the cytoplasm and the intracellular pH value decreased [87]. Among them, lysosomal pathway with cathepsin-B activity simultaneously mediates tumor cell death [88]. In addition, the external alternating magnetic field can also cause the Neil relaxation loss of magnetic nanoparticles to generate heat. This causes the temperature in the tumor tissue to rise to more than 43 °C, causing cancer cell necrosis [89]. To sum up, this opens up new opportunities for cancer treatment.

## Effect of IONPs on EVs

EVs are small vesicles secreted by various cells. They can carry a variety of signal biomolecules, including protein, mRNA and microRNA. In the past decade, EVs derived from stem cells have become promising tools for information transmission, treatment and marker detection in medical research. EVs are produced by budding from the inner chamber or plasma membrane and are composed of exosomes (50–150 nm) and microvesicles. In particular, the study of exosomes has attracted more and more attention in the fields of cancer and tissue regeneration due to their unique biological characteristics. However, the low separation rate and insufficient targeting ability limit their therapeutic applicability. In order to understand the regeneration mechanism caused by stem cell-derived EVs, researchers introduced magnetic particles. SPION with magnetic navigation can enhance the aiming ability of EVs.

### Effect of IONPs on EVs generation

EVs are produced or released spontaneously in response to biological, chemical or physical triggers during cell culture [90]. Therefore, cells can release magnetic vesicles when they are co-incubated with IONPs [91]. Magnetic labeling of extracellular vesicles derived from stem cells for MRI imaging [92]. The experiment shows that Fe_3_O_4_ magnetic nanoparticles with a certain dose and exposure times can enhance cell proliferation when co-incubated with cells [12, 34]. In addition, the cells treated with IONPs show high activity, and the nanoparticles may stimulate the biogenesis of EVs. However, this situation is different in different cell types. In an experiment to study the transmission of magnetic EVs to the mouse brain, Kutchy et al. [93] It was found that USPIO had no significant effect on the release of astrocytes and EVs. The reason and potential mechanism for the increase of EVs particle concentration are still unclear. However, the mRNA expression of some vesicle-associated transporters has changed [12]. In conclusion, the uptake of IONPs by cells can enhance the secretion of EVs. This can improve the problem of low secretion productivity.

The labeled cells will lose magnetic nanoparticles when pressed, resulting in a decrease in the magnetic properties of the donor cells. In addition, microvesicles are released and transferred to other cells of the host organism through the mechanism of cross-transfer between cells [18]. Therefore, after internalization by monocytes or macrophages, magnetic nanoparticles can be released by cells in the form of vesicles under pressure or activation [94]. These vesicles are absorbed by immature macrophages and trigger magnetic labeled intercellular transfer. During the differentiation of magnetically labeled monocytes into macrophages, they also lead to the redistribution of magnetic markers. Silver et al. [95] also found that the microvesicles shed by stress cells were involved in the redistribution of magnetic labeled immature cells. They were detected by MRI.

### Effect of IONPs on EVs separation

The study of exosomes is of great significance to the diagnosis and prognosis of cancer and has potential in the medical field. The noninvasive separation of exosomes is a crucial step because their concentration in complex body fluids is extremely low and their volume is small [96]. Therefore, it is very important to obtain a good recovery rate and maintain the physical properties for the diagnosis and treatment of cancer [97]. The introduction of magnetic nanoparticles can improve the delivery efficiency of exosomes, thereby improving the therapeutic effect and reducing side effects. At the same time, it can provide a new method for rapid, efficient and high purity exosome separation in cell culture supernatants or complex biological fluids. EVs were isolated and captured from different clinical samples using an external magnetic field, maintaining their biological integrity and further facilitating the application of exosomes in the required fields.

At present, the methods used to separate EVs include ultracentrifugation (UC), electroporation, magnetoacoustic perforation, magnetic sorting and immunoaffinity capture. However, the integrity of EVs can be damaged if magnetic particles are loaded by electroporation and ultrasound. This direct loading method can be considered if the EVs are only used to transport drugs. There is no special requirement for EV-specific endogenous substances. Another top-down method is to produce magnetic EVs by simultaneously incubating cells and magnetic nanoparticles. EVs are separated through UC. However, ultrahigh-speed centrifugal force may cause EV fragmentation, cargo leakage and protein sedimentation. Therefore, to avoid damage to EVs, magnetic separation can be used [90, 98]. This method involves absorption by a magnet, which is relatively mild. Guo et al. [99] prepared exosome mimics (EM) directly by magnetic extrusion after the cells engulfed the magnetic particles. EM has the same biological origin, composition, biological functions and similar morphology as natural exosomes. The main advantage of the magnetic extrusion method is the ability to harvest a large number of therapeutic vesicles. In addition, immune affinity capture can be selected. EVs carry various biomarkers. The magnetic nanoparticles are modified to form immunoaffinity magnetic nanoparticles [100–103]. Then, the exosome markers (CD63, CD9 and CD81) [104] can be recognized and combined, effectively separated and released with high efficiency [105–108].

For example, Brambilla et al. [97] immobilized DNA based on an anti-CD63 antibody and released EVs by using the endonuclease activity of DNAse I. Zhang et al. [109] rapidly separated microvesicles from the supernatant of cells by streptomycin-modified iron oxide nanoparticles (SA-IONPs). In addition, blood is one of the most commonly collected biological fluids [108]. For example, María et al. [110] successfully used an exosome-binding antibody (anti-CD9) to functionalize magnetic nanoparticles to capture exosomes from the whole blood of pancreatic cancer patients. Cai et al. [96] successfully functionalized IONPs with exosome-binding antibodies (anti-CD9) and separated exosomes from whole blood through a new microfluidic device. Liu et al. [111] modified IONPs with the antibody CD63 to bind with an antigen and capture exosomes. Yang et al. [112] showed that superparamagnetic nanoparticles labeled with transferrin (Tfs) can accurately bind to blood TfR^+^ exosomes. In addition, Chang et al. [113] removed the contamination of soluble proteins (such as albumin and fibrinogen) and lipoprotein particles to achieve the purification of exosomes by coating IONPs with the network structure of PEG. Moreover, compared with the exosomes separated by classical ultracentrifugation, the exosomes separated and released by continuous immunomagnetic separation maintained structural integrity and good biological activity.

In summary, only high concentrations of EVs can ensure the analysis of downstream proteins and nucleic acid components. Therefore, we discuss the methods of exosome isolation currently used. Different extraction methods have their own advantages and disadvantages. Researchers should select appropriate IONPs methods to highly enrich and purify EVs according to specific conditions and constantly optimize the separation method.

### Therapeutic of the magnetic EVs

Magnetic targeting drug delivery systems are of great significance in biomedical applications, as they are used to improve therapeutic efficiency. IONPs have also proven to be an effective drug delivery platform for both BBB and ischemic stroke treatment. These EVs are designed to wrap different therapeutic agents and iron oxide nanoparticles together. These magnetic vesicles can perform dynamic regulation and spatial control under a magnetic field [114], enrich at the target location, enhance cell uptake, and thus enhance the therapeutic effect of EVs [115].

MiRNA is one of the main functional components of exosomes, which may play a key role in cell communication and ultimately the regulation of biological functions. Wu et al. [116] revealed that the abundance of miR-1260a in exosomes released by BMSCs pretreated with Fe_3_O_4_ nanoparticles and SMFs was greatly increased. At the same time, the upregulation of miR-21-5p in mag-BMSC-Exos may promote wound healing [117] and tendon bone healing through the miR-21-5p/SMAD7 pathway [118]. In a heart repair experiment, Lee et al. [119] found that the NVs of MSCs derived from IONPs (IONP NVs) contain more heart repair therapeutic molecules (RNA and proteins) than normal MSC-derived NVs, which are mediated by the intracellular signal transduction modification triggered by the incorporation and ionization of iron oxide. With the slow ionization and assimilation of IONPs in vivo [120], the treatment of MSCs by IONP can improve the expression of anti-inflammatory and tissue repair cytokines by activating the JNK signal pathway [119, 121, 122]. At the same time, the level of therapeutic molecules in the prepared magnetic EVs increased. In the treatment of infarcted heart, IONP up-regulates the therapeutic molecules in IONP-MSC, including ANG1, FGF2, HGF, VEGF and Cx43, and these up-regulated molecules are also retained in IONP-NV [119]. In addition, exosomes coated with magnetic nanoparticles can be enriched at the tumor site under the guidance of an external magnetic field. The application of near-infrared radiation will cause local hyperthermia and trigger the release of goods loaded in the exosome [123, 124], thus enhancing the therapeutic effect [125].

In summary, we reviewed the influence of internalized IONPs on the therapeutic mechanism of EVs. In particular, we investigated whether IONP-Exos have a more significant therapeutic effect than normal exosomes. Although some achievements have been made, more experimental evidence is still sought to prove this effect.

## Conclusion and prospects

In this paper, we reviewed the way in which IONPs were absorbed by cells and the mechanism of transport, degradation and toxicity of IONPs in cells. Therefore, IONPs can exert positive effects on cells by controlling the dosage. But it can also kill tumor cells. In stem cell therapy, the introduction of IONPs will make the ionization of IONPs trigger more therapeutic molecules (RNA and protein) for repair. Therefore, the fate of IONPs in cells is fully described to further promote the application of magnetic nanomaterials in the biomedical field. Extracellular vesicles have been proved to be suitable for the treatment of various diseases in vitro and in vivo. On this basis, the introduction of magnetic particles presents various research opportunities and challenges for the field of nanomedicine. In addition, this article briefly introduces the effects of IONPs on the production, separation and treatment mechanism of EVs. Most of the current work involves the combination of magnetic vesicles and magnetic fields to provide tissue-specific EVs delivery for therapeutic applications. However, the introduction of magnetic particles will also induce the triggering and release of cell therapeutic factors in EVs to achieve more effective disease treatment. However, more data are needed to prove the impact of IONPs on cells and EVs.

This overview aims to solve the existing problems and fully understand the effect of IONPs on cells and EVs delivery system. It is still necessary to further study the effect of IONPs on cells and EVs at multiple levels to better understand their mechanism of action and ensure the proper use of IONPs in the field of precision medicine. The therapeutic application of EVs based on IONPs also has high clinical conversion potential. Therefore, further efforts are needed in the future.

## Data Availability

The data used to support the findings of this study are available from the corresponding author upon request.

## References

[1] Mahmoudi M, Hosseinkhani H, Hosseinkhani M, Boutry S, Simchi A, Journeay WS (2011). Magnetic resonance imaging tracking of stem cells in vivo using iron oxide nanoparticles as a tool for the advancement of clinical regenerative medicine. Chem Rev.

[2] Laurent S, Forge D, Port M, Roch A, Robic C, Vander Elst L (2008). Magnetic iron oxide nanoparticles: synthesis, stabilization, vectorization, physicochemical characterizations, and biological applications. Chem Rev.

[3] Abd Elkodous M, El-Sayyad GS, Abdelrahman IY, El-Bastawisy HS, Mohamed AE, Mosallam FM (2019). Therapeutic and diagnostic potential of nanomaterials for enhanced biomedical applications. Colloids Surf B Biointerfaces.

[4] Hu Y, Mignani S, Majoral J-P, Shen M, Shi X (2018). Construction of iron oxide nanoparticle-based hybrid platforms for tumor imaging and therapy. Chem Soc Rev.

[5] Ahmadi M, Elmongy H, Madrakian T, Abdel-Rehim M (2017). Nanomaterials as sorbents for sample preparation in bioanalysis: a review. Anal Chim Acta.

[6] Pershina AG, Brikunova OY, Demin AM, Shevelev OB, Razumov IA, Zavjalov EL (2020). pH-triggered delivery of magnetic nanoparticles depends on tumor volume. Nanomedicine.

[7] Demin AM, Mekhaev AV, Kandarakov OF, Popenko VI, Leonova OG, Murzakaev AM (2020). L-Lysine-modified FeO nanoparticles for magnetic cell labeling. Colloids Surf B Biointerfaces.

[8] Arami H, Khandhar A, Liggitt D, Krishnan KM (2015). In vivo delivery, pharmacokinetics, biodistribution and toxicity of iron oxide nanoparticles. Chem Soc Rev.

[9] Sart S, Bejarano FC, Baird MA, Yan Y, Rosenberg JT, Ma T (2015). Intracellular labeling of mouse embryonic stem cell-derived neural progenitor aggregates with micron-sized particles of iron oxide. Cytotherapy.

[10] Zhuo Z, Wang J, Luo Y, Zeng R, Zhang C, Zhou W (2021). Targeted extracellular vesicle delivery systems employing superparamagnetic iron oxide nanoparticles. Acta Biomater.

[11] Yarjanli Z, Ghaedi K, Esmaeili A, Rahgozar S, Zarrabi A (2017). Iron oxide nanoparticles may damage to the neural tissue through iron accumulation, oxidative stress, and protein aggregation. BMC Neurosci.

[12] Marzano M, Bou-Dargham MJ, Cone AS, York S, Helsper S, Grant SC (2021). Biogenesis of extracellular vesicles produced from human-stem-cell-derived cortical spheroids exposed to iron oxides. ACS Biomater Sci Eng.

[13] Rascol E, Daurat M, Da Silva A, Maynadier M, Dorandeu C, Charnay C (2017). Biological fate of Fe_3_O_4_ core-shell mesoporous silica nanoparticles depending on particle surface Chemistry. Nanomaterials (Basel).

[14] Hofmann D, Tenzer S, Bannwarth MB, Messerschmidt C, Glaser S-F, Schild H (2014). Mass spectrometry and imaging analysis of nanoparticle-containing vesicles provide a mechanistic insight into cellular trafficking. ACS Nano.

[15] Arsianti M, Lim M, Marquis CP, Amal R (2010). Polyethylenimine based magnetic iron-oxide vector: the effect of vector component assembly on cellular entry mechanism, intracellular localization, and cellular viability. Biomacromolecules.

[16] Portilla Y, Mellid S, Paradela A, Ramos-Fernández A, Daviu N, Sanz-Ortega L (2021). Iron oxide nanoparticle coatings dictate cell outcomes despite the influence of protein coronas. ACS Appl Mater Interfaces.

[17] Portilla Y, Mulens-Arias V, Paradela A, Ramos-Fernández A, Pérez-Yagüe S, Morales MP (2022). The surface coating of iron oxide nanoparticles drives their intracellular trafficking and degradation in endolysosomes differently depending on the cell type. Biomaterials.

[18] Lunov O, Syrovets T, Büchele B, Jiang X, Röcker C, Tron K (2010). The effect of carboxydextran-coated superparamagnetic iron oxide nanoparticles on c-Jun N-terminal kinase-mediated apoptosis in human macrophages. Biomaterials.

[19] Wu H-Y, Chung M-C, Wang C-C, Huang C-H, Liang H-J, Jan T-R (2013). Iron oxide nanoparticles suppress the production of IL-1beta via the secretory lysosomal pathway in murine microglial cells. Part Fibre Toxicol.

[20] Arbab AS, Wilson LB, Ashari P, Jordan EK, Lewis BK, Frank JA (2005). A model of lysosomal metabolism of dextran coated superparamagnetic iron oxide (SPIO) nanoparticles: implications for cellular magnetic resonance imaging. NMR Biomed.

[21] Chen Y-C, Hsiao J-K, Liu H-M, Lai IY, Yao M, Hsu S-C (2010). The inhibitory effect of superparamagnetic iron oxide nanoparticle (Ferucarbotran) on osteogenic differentiation and its signaling mechanism in human mesenchymal stem cells. Toxicol Appl Pharm.

[22] Singh N, Jenkins GJS, Asadi R, Doak SH. Potential toxicity of superparamagnetic iron oxide nanoparticles (SPION). Nano Rev. 2010;1:5358-15. 10.3402/nano.v1i0.5358 PMC3215220 22110864

[23] Ghosh S, Ghosh I, Chakrabarti M, Mukherjee A (2020). Genotoxicity and biocompatibility of superparamagnetic iron oxide nanoparticles: Influence of surface modification on biodistribution, retention, DNA damage and oxidative stress. Food Chem Toxicol.

[24] Cairo G, Recalcati S, Mantovani A, Locati M (2011). Iron trafficking and metabolism in macrophages: contribution to the polarized phenotype. Trends Immunol.

[25] Vela D (2018). Iron metabolism in prostate cancer; from basic science to new therapeutic strategies. Front Oncol.

[26] Xie Y, Liu D, Cai C, Chen X, Zhou Y, Wu L (2016). Size-dependent cytotoxicity of Fe3O4 nanoparticles induced by biphasic regulation of oxidative stress in different human hepatoma cells. Int J Nanomed.

[27] Chen S, Chen S, Zeng Y, Lin L, Wu C, Ke Y (2018). Size-dependent superparamagnetic iron oxide nanoparticles dictate interleukin-1β release from mouse bone marrow-derived macrophages. J Appl Toxicol.

[28] Diaz-Diestra DM, Palacios-Hernandez T, Liu Y, Smith DE, Nguyen AK, Todorov T (2022). Impact of surface Chemistry of ultrasmall superparamagnetic iron oxide nanoparticles on protein corona formation and endothelial cell uptake, toxicity, and barrier function. Toxicol Sci.

[29] Gupta AK, Gupta M (2005). Cytotoxicity suppression and cellular uptake enhancement of surface modified magnetic nanoparticles. Biomaterials.

[30] Rafieepour A, Azari MR, Peirovi H, Khodagholi F, Jaktaji JP, Mehrabi Y (2019). Investigation of the effect of magnetite iron oxide particles size on cytotoxicity in A549 cell line. Toxicol Ind Health.

[31] Janik-Olchawa N, Drozdz A, Ryszawy D, Pudelek M, Planeta K, Setkowicz Z (2021). The influence of IONPs core size on their biocompatibility and activity in in vitro cellular models. Sci Rep.

[32] Patil US, Adireddy S, Jaiswal A, Mandava S, Lee BR, Chrisey DB (2015). In vitro/in vivo toxicity evaluation and quantification of iron oxide nanoparticles. Int J Mol Sci.

[33] Vakili-Ghartavol R, Momtazi-Borojeni AA, Vakili-Ghartavol Z, Aiyelabegan HT, Jaafari MR, Rezayat SM (2020). Toxicity assessment of superparamagnetic iron oxide nanoparticles in different tissues. Artif Cells Nanomed Biotechnol.

[34] Janik-Olchawa N, Drozdz A, Ryszawy D, Pudełek M, Planeta K, Setkowicz Z (2020). Comparison of ultrasmall IONPs and Fe salts biocompatibility and activity in multi-cellular in vitro models. Sci Rep.

[35] Yan Y, Sart S, Calixto Bejarano F, Muroski ME, Strouse GF, Grant SC (2015). Cryopreservation of embryonic stem cell-derived multicellular neural aggregates labeled with micron-sized particles of iron oxide for magnetic resonance imaging. Biotechnol Prog.

[36] Ferraz FS, López JL, Lacerda SMSN, Procópio MS, Figueiredo AFA, Martins EMN (2020). Biotechnological approach to induce human fibroblast apoptosis using superparamagnetic iron oxide nanoparticles. J Inorg Biochem.

[37] Valdiglesias V, Kiliç G, Costa C, Fernández-Bertólez N, Pásaro E, Teixeira JP (2015). Effects of iron oxide nanoparticles: cytotoxicity, genotoxicity, developmental toxicity, and neurotoxicity. Environ Mol Mutagen.

[38] Liu Y, Li J, Xu K, Gu J, Huang L, Zhang L (2018). Characterization of superparamagnetic iron oxide nanoparticle-induced apoptosis in PC12 cells and mouse hippocampus and striatum. Toxicol Lett.

[39] Zanganeh S, Hutter G, Spitler R, Lenkov O, Mahmoudi M, Shaw A (2016). Iron oxide nanoparticles inhibit tumour growth by inducing pro-inflammatory macrophage polarization in tumour tissues. Nat Nanotechnol.

[40] Jin R, Liu L, Zhu W, Li D, Yang L, Duan J (2019). Iron oxide nanoparticles promote macrophage autophagy and inflammatory response through activation of toll-like Receptor-4 signaling. Biomaterials.

[41] Peynshaert K, Manshian BB, Joris F, Braeckmans K, De Smedt SC, Demeester J (2014). Exploiting intrinsic nanoparticle toxicity: the pros and cons of nanoparticle-induced autophagy in biomedical research. Chem Rev.

[42] Zhou X, Jin W, Sun H, Li C, Jia J (2022). Perturbation of autophagy: an intrinsic toxicity mechanism of nanoparticles. Sci Total Environ.

[43] Bulte JWM (2009). In vivo MRI cell tracking: clinical studies. AJR Am J Roentgenol.

[44] Kostura L, Kraitchman DL, Mackay AM, Pittenger MF, Bulte JWM (2004). Feridex labeling of mesenchymal stem cells inhibits chondrogenesis but not adipogenesis or osteogenesis. NMR Biomed.

[45] Farrell E, Wielopolski P, Pavljasevic P, van Tiel S, Jahr H, Verhaar J (2008). Effects of iron oxide incorporation for long term cell tracking on MSC differentiation in vitro and in vivo. Biochem Biophys Res Commun.

[46] Huang D-M, Hsiao J-K, Chen Y-C, Chien L-Y, Yao M, Chen Y-K (2009). The promotion of human mesenchymal stem cell proliferation by superparamagnetic iron oxide nanoparticles. Biomaterials.

[47] Li X, Wei Z, Lv H, Wu L, Cui Y, Yao H (2019). Iron oxide nanoparticles promote the migration of mesenchymal stem cells to injury sites. Int J Nanomed.

[48] Li X, Wei Z, Li B, Li J, Lv H, Wu L (2019). In vivo migration of Fe3O4@polydopamine nanoparticle-labeled mesenchymal stem cells to burn injury sites and their therapeutic effects in a rat model. Biomater Sci.

[49] Huang X, Zhang F, Wang Y, Sun X, Choi KY, Liu D (2014). Design considerations of iron-based nanoclusters for noninvasive tracking of mesenchymal stem cell homing. ACS Nano.

[50] Yun WS, Choi JS, Ju HM, Kim MH, Choi SJ, Oh ES, et al. Enhanced homing technique of mesenchymal stem cells using iron oxide nanoparticles by magnetic attraction in olfactory-injured mouse models. Int J Mol Sci. 2018;19:1376-16. 10.3390/ijms19051376 PMC5983763 29734748

[51] Arbab AS, Jordan EK, Wilson LB, Yocum GT, Lewis BK, Frank JA (2004). In vivo trafficking and targeted delivery of magnetically labeled stem cells. Hum Gene Ther.

[52] Schulze F, Gramoun A, Crowe LA, Dienelt A, Akcan T, Hofmann H (2015). Accumulation of amino-polyvinyl alcohol-coated superparamagnetic iron oxide nanoparticles in bone marrow: implications for local stromal cells. Nanomed (Lond).

[53] Schulze F, Dienelt A, Geissler S, Zaslansky P, Schoon J, Henzler K (2014). Amino-polyvinyl alcohol coated superparamagnetic iron oxide nanoparticles are suitable for monitoring of human mesenchymal stromal cells in vivo. Small (Weinh Der Bergstr, Ger).

[54] Jiang P, Zhang Y, Zhu C, Zhang W, Mao Z, Gao C (2016). Fe3O4/BSA particles induce osteogenic differentiation of mesenchymal stem cells under static magnetic field. Acta Biomater.

[55] Andreas K, Georgieva R, Ladwig M, Mueller S, Notter M, Sittinger M (2012). Highly efficient magnetic stem cell labeling with citrate-coated superparamagnetic iron oxide nanoparticles for MRI tracking. Biomaterials.

[56] Bulte JWM, Kraitchman DL, Mackay AM, Pittenger MF. Chondrogenic differentiation of mesenchymal stem cells is inhibited after magnetic labeling with ferumoxides. Blood. 2004;104:3410-2. 10.1182/blood-2004-06-2117 15525839

[57] Han J, Kim B, Shin J-Y, Ryu S, Noh M, Woo J (2015). Iron oxide nanoparticle-mediated development of cellular gap junction crosstalk to improve mesenchymal stem cells’ therapeutic efficacy for myocardial infarction. ACS Nano.

[58] Huang T, Zhang T, Jiang X, Li A, Su Y, Bian Q (2021). Iron oxide nanoparticles augment the intercellular mitochondrial transfer-mediated therapy. Sci Adv.

[59] Yun S, Shin T-H, Lee J-H, Cho MH, Kim I-S, Kim J-W (2018). Design of magnetically labeled cells (mag-cells) for in vivo control of stem cell migration and differentiation. Nano Lett.

[60] Duan X, Li Y (2013). Physicochemical characteristics of nanoparticles affect circulation, biodistribution, cellular internalization, and trafficking. Small (Weinh Der Bergstr, Ger).

[61] Yun WS, Aryal S, Ahn YJ, Seo YJ, Key J (2020). Engineered iron oxide nanoparticles to improve regenerative effects of mesenchymal stem cells. Biomed Eng Lett.

[62] Liang X, Chen M, Bhattarai P, Hameed S, Tang Y, Dai Z (2021). Complementing cancer photodynamic therapy with ferroptosis through iron oxide loaded porphyrin-grafted lipid nanoparticles. ACS Nano.

[63] Rojas JM, Sanz-Ortega L, Mulens-Arias V, Gutiérrez L, Pérez-Yagüe S, Barber DF (2016). Superparamagnetic iron oxide nanoparticle uptake alters M2 macrophage phenotype, iron metabolism, migration and invasion. Nanomedicine.

[64] Ying H, Ruan Y, Zeng Z, Bai Y, Xu J, Chen S (2022). Iron oxide nanoparticles size-dependently activate mouse primary macrophages via oxidative stress and endoplasmic reticulum stress. Int Immunopharmacol.

[65] Laskar A, Eilertsen J, Li W, Yuan X-M (2013). SPION primes THP1 derived M2 macrophages towards M1-like macrophages. Biochem Biophys Res Commun.

[66] Wu C, Shen Z, Lu Y, Sun F, Shi H (2022). p53 Promotes ferroptosis in macrophages treated with Fe3O4 nanoparticles. ACS Appl Mater Interfaces.

[67] Zhu L, Wang J, Tang X, Zhang C, Wang P, Wu L (2022). Efficient magnetic nanocatalyst-induced chemo- and ferroptosis synergistic cancer therapy in combination with t1-t2 dual-mode magnetic resonance imaging through doxorubicin delivery. ACS Appl Mater Interfaces.

[68] Khan MI, Mohammad A, Patil G, Naqvi SAH, Chauhan LKS, Ahmad I (2012). Induction of ROS, mitochondrial damage and autophagy in lung epithelial cancer cells by iron oxide nanoparticles. Biomaterials.

[69] Luo K, Zhao J, Jia C, Chen Y, Zhang Z, Zhang J (2020). Integration of Fe3O4 with Bi2S3 for multi-modality tumor theranostics. ACS Appl Mater Interfaces.

[70] Wu H, Xing H, Wu M-C, Shen F, Chen Y, Yang T (2021). Extracellular-vesicles delivered tumor-specific sequential nanocatalysts can be used for MRI-informed nanocatalytic Therapy of hepatocellular carcinoma. Theranostics.

[71] Lin L-S, Huang T, Song J, Ou X-Y, Wang Z, Deng H (2019). Synthesis of copper peroxide nanodots for H2O2 self-supplying chemodynamic therapy. J Am Chem Soc.

[72] Ma PA, Xiao H, Yu C, Liu J, Cheng Z, Song H (2017). Enhanced cisplatin chemotherapy by iron oxide nanocarrier-mediated generation of highly toxic reactive oxygen species. Nano Lett.

[73] Feng L, Xie R, Wang C, Gai S, He F, Yang D (2018). Magnetic targeting, tumor microenvironment-responsive intelligent nanocatalysts for enhanced tumor ablation. ACS Nano.

[74] Gao Z, He T, Zhang P, Li X, Zhang Y, Lin J (2020). Polypeptide-based theranostics with tumor-microenvironment-activatable cascade reaction for chemo-ferroptosis combination therapy. ACS Appl Mater Interfaces.

[75] Liu Y, Quan X, Li J, Huo J, Li X, Zhao Z (2023). Liposomes embedded with PEGylated iron oxide nanoparticles enable ferroptosis and combination therapy in cancer. Natl Sci Rev.

[76] Shen Z, Liu T, Li Y, Lau J, Yang Z, Fan W (2018). Fenton-reaction-acceleratable magnetic nanoparticles for ferroptosis therapy of orthotopic brain tumors. ACS Nano.

[77] Xie S, Sun W, Zhang C, Dong B, Yang J, Hou M (2021). Metabolic control by heat stress determining cell fate to ferroptosis for effective cancer therapy. ACS Nano.

[78] Wahajuddin, Arora S (2012). Superparamagnetic iron oxide nanoparticles: magnetic nanoplatforms as drug carriers. Int J Nanomed.

[79] Semeano AT, Tofoli FA, Corrêa-Velloso JC, de Jesus Santos AP, Oliveira-Giacomelli Á, Cardoso RR (2022). Effects of magnetite nanoparticles and static magnetic field on neural differentiation of pluripotent stem cells. Stem Cell Rev Rep.

[80] Watanabe M, Yoneda M, Morohashi A, Hori Y, Okamoto D, Sato A (2013). Effects of Fe3O4 magnetic nanoparticles on A549 cells. Int J Mol Sci.

[81] Gao J, Zhou H, Zhao Y, Lu L, Zhang J, Cheng W (2021). Time-course effect of ultrasmall superparamagnetic iron oxide nanoparticles on intracellular iron metabolism and ferroptosis activation. Nanotoxicology.

[82] Zhang Y, Xia M, Zhou Z, Hu X, Wang J, Zhang M (2021). p53 Promoted ferroptosis in ovarian cancer cells treated with human serum incubated-superparamagnetic iron oxides. Int J Nanomed.

[83] Zhou H, Choi SI, Zou F, Oh S, Kim JE, Hwang DY (2014). Cytotoxicity and gene expression in sarcoma 180 cells in response to spiky magnetoplasmonic supraparticles. ACS Appl Mater Interfaces.

[84] Dobson J (2008). Remote control of cellular behaviour with magnetic nanoparticles. Nat Nanotechnol.

[85] Corchero JL, Villaverde A (2009). Biomedical applications of distally controlled magnetic nanoparticles. Trends Biotechnol.

[86] Lunov O, Uzhytchak M, Smolková B, Lunova M, Jirsa M, Dempsey NM, et al. Remote actuation of apoptosis in liver cancer cells via magneto-mechanical modulation of iron oxide nanoparticles. Cancers (Basel). 2019;11:1873-21. 10.3390/cancers11121873 PMC6966689 31779223

[87] Zhang E, Kircher MF, Koch M, Eliasson L, Goldberg SN, Renström E (2014). Dynamic magnetic fields remote-control apoptosis via nanoparticle rotation. ACS Nano.

[88] Lopez S, Hallali N, Lalatonne Y, Hillion A, Antunes JC, Serhan N (2022). Magneto-mechanical destruction of cancer-associated fibroblasts using ultra-small iron oxide nanoparticles and low frequency rotating magnetic fields. Nanoscale Adv.

[89] Kobayashi T (2011). Cancer hyperthermia using magnetic nanoparticles. Biotechnol J.

[90] Piffoux M, Silva AKA, Lugagne J-B, Hersen P, Wilhelm C, Gazeau F (2017). Extracellular vesicle production loaded with nanoparticles and drugs in a trade-off between loading, yield and purity: towards a personalized drug delivery system. Adv Biosyst.

[91] Kang K, Zhou X, Zhang Y, Zhu N, Li G, Yi Q (2021). Cell-released magnetic vesicles capturing metabolic labeled rare circulating tumor cells based on bioorthogonal Chemistry. Small (Weinh Der Bergstr, Ger).

[92] Dabrowska S, Del Fattore A, Karnas E, Frontczak-Baniewicz M, Kozlowska H, Muraca M (2018). Imaging of extracellular vesicles derived from human bone marrow mesenchymal stem cells using fluorescent and magnetic labels. Int J Nanomed.

[93] Kutchy NA, Ma R, Liu Y, Buch S, Hu G (2022). Extracellular vesicle-mediated delivery of ultrasmall superparamagnetic iron oxide nanoparticles to mice brain. Front Pharm.

[94] Luciani N, Wilhelm C, Gazeau F (2010). The role of cell-released microvesicles in the intercellular transfer of magnetic nanoparticles in the monocyte/macrophage system. Biomaterials.

[95] Silva AKA, Wilhelm C, Kolosnjaj-Tabi J, Luciani N, Gazeau F (2012). Cellular transfer of magnetic nanoparticles via cell microvesicles: impact on cell tracking by magnetic resonance imaging. Pharm Res.

[96] Cai S, Luo B, Jiang P, Zhou X, Lan F, Yi Q (2018). Immuno-modified superparamagnetic nanoparticles via host-guest interactions for high-purity capture and mild release of exosomes. Nanoscale.

[97] Brambilla D, Sola L, Ferretti AM, Chiodi E, Zarovni N, Fortunato D (2021). EV separation: release of intact extracellular vesicles immunocaptured on magnetic particles. Anal Chem.

[98] Doyle LM, Wang MZ. Overview of extracellular vesicles, their origin, composition, purpose, and methods for exosome isolation and analysis. Cells. 2019;8:727. 10.3390/cells8070727 PMC6678302 31311206

[99] Guo P, Busatto S, Huang J, Morad G, Moses MA. A facile magnetic extrusion method for preparing endosome-derived vesicles for cancer drug delivery. Adv Funct Mater. 2021;31:2008326. 10.1002/adfm.202008326 PMC8680268 34924915

[100] Takov K, Yellon DM, Davidson SM (2019). Comparison of small extracellular vesicles isolated from plasma by ultracentrifugation or size-exclusion chromatography: yield, purity and functional potential. J Extracell Vesicles.

[101] Kang Y-T, Hadlock T, Lo T-W, Purcell E, Mutukuri A, Fouladdel S (2020). Dual-Isolation and profiling of circulating tumor cells and cancer exosomes from blood samples with melanoma using immunoaffinity-based microfluidic interfaces. Adv Sci (Weinh).

[102] Sharma P, Ludwig S, Muller L, Hong CS, Kirkwood JM, Ferrone S (2018). Immunoaffinity-based isolation of melanoma cell-derived exosomes from plasma of patients with melanoma. J Extracell Vesicles.

[103] Shi L, Cao J, Yang C, Wang X, Shi K, Shang L (2022). Hierarchical magnetic nanoparticles for highly effective capture of small extracellular vesicles. J Colloid Interface Sci.

[104] Zhang W, Lu R, Zhang L. Preparation of dual-functional composite magnetic nanomaterials modified with different metals/aptamers and their performance in exosome enrichment. Se Pu. 2021;39:1128–36. 10.3724/SP.J.1123.2021.06012 PMC9404067 34505435

[105] Cheng J, Zhu N, Zhang Y, Yu Y, Kang K, Yi Q (2022). Hedgehog-inspired immunomagnetic beads for high-efficient capture and release of exosomes. J Mater Chem B.

[106] Zhu N, Zhang Y, Cheng J, Mao Y, Kang K, Li G (2022). Immuno-affinitive supramolecular magnetic nanoparticles incorporating cucurbit[8]uril-mediated ternary host-guest complexation structures for high-efficient small extracellular vesicle enrichment. J Colloid Interface Sci.

[107] Zhang Y, Chen L, Ye X, Wu Z, Zhang Z, Sun B (2021). Expression and mechanism of exosome-mediated A FOXM1 related long noncoding RNA in gastric cancer. J Nanobiotechnol.

[108] Karimi N, Dalirfardouei R, Dias T, Lötvall J, Lässer C (2022). Tetraspanins distinguish separate extracellular vesicle subpopulations in human serum and plasma - Contributions of platelet extracellular vesicles in plasma samples. J Extracell Vesicles.

[109] Zhang W, Yu Z-L, Wu M, Ren J-G, Xia H-F, Sa G-L (2017). Magnetic and folate functionalization enables rapid isolation and enhanced tumor-targeting of cell-derived microvesicles. ACS Nano.

[110] Sancho-Albero M, Sebastián V, Sesé J, Pazo-Cid R, Mendoza G, Arruebo M (2020). Isolation of exosomes from whole blood by a new microfluidic device: proof of concept application in the diagnosis and monitoring of pancreatic cancer. J Nanobiotechnol.

[111] Liu S, Chen X, Bao L, Liu T, Yuan P, Yang X (2020). Treatment of infarcted heart tissue via the capture and local delivery of circulating exosomes through antibody-conjugated magnetic nanoparticles. Nat Biomed Eng.

[112] Yang L, Han D, Zhan Q, Li X, Shan P, Hu Y (2019). Blood TfR+ exosomes separated by a pH-responsive method deliver chemotherapeutics for tumor therapy. Theranostics.

[113] Chang M, Chang Y-J, Chao PY, Yu Q (2018). Exosome purification based on PEG-coated Fe3O4 nanoparticles. PloS One.

[114] Silva AKA, Luciani N, Gazeau F, Aubertin K, Bonneau S, Chauvierre C (2015). Combining magnetic nanoparticles with cell derived microvesicles for drug loading and targeting. Nanomedicine.

[115] Zhang J, Ji C, Zhang H, Shi H, Mao F, Qian H (2022). Engineered neutrophil-derived exosome-like vesicles for targeted cancer therapy. Sci Adv.

[116] Wu D, Chang X, Tian J, Kang L, Wu Y, Liu J (2021). Bone mesenchymal stem cells stimulation by magnetic nanoparticles and a static magnetic field: release of exosomal miR-1260a improves osteogenesis and angiogenesis. J Nanobiotechnol.

[117] Wu D, Kang L, Tian J, Wu Y, Liu J, Li Z (2020). Exosomes derived from bone mesenchymal stem cells with the stimulation of FeO nanoparticles and static magnetic field enhance wound healing through upregulated miR-21-5p. Int J Nanomed.

[118] Wu X-D, Kang L, Tian J, Wu Y, Huang Y, Liu J (2022). Exosomes derived from magnetically actuated bone mesenchymal stem cells promote tendon-bone healing through the miR-21-5p/SMAD7 pathway. Mater Today Bio.

[119] Lee J-R, Park B-W, Kim J, Choo YW, Kim HY, Yoon J-K (2020). Nanovesicles derived from iron oxide nanoparticles-incorporated mesenchymal stem cells for cardiac repair. Sci Adv.

[120] Kolosnjaj-Tabi J, Lartigue L, Javed Y, Luciani N, Pellegrino T, Wilhelm C, et al. Biotransformations of magnetic nanoparticles in the body. Nano Today. 2016;11:280–4.

[121] Kim HY, Kumar H, Jo M-J, Kim J, Yoon J-K, Lee J-R (2018). Therapeutic efficacy-potentiated and diseased organ-targeting nanovesicles derived from mesenchymal stem cells for spinal cord injury treatment. Nano Lett.

[122] Jung M, Kim H, Hwang JW, Choi Y, Kang M, Kim C (2023). Iron oxide nanoparticle-incorporated mesenchymal stem cells for Alzheimer’s disease treatment. Nano Lett.

[123] Wang J, Chen P, Dong Y, Xie H, Wang Y, Soto F (2021). Designer exosomes enabling tumor targeted efficient chemo/gene/photothermal therapy. Biomaterials.

[124] Kwon S-H, Faruque HA, Kee H, Kim E, Park S (2021). Exosome-based hybrid nanostructures for enhanced tumor targeting and hyperthermia therapy. Colloids Surf B Biointerfaces.

[125] Zheng D, Wan C, Yang H, Xu L, Dong Q, Du C (2020). Her2-targeted multifunctional nano-theranostic platform mediates tumor microenvironment remodeling and immune activation for breast cancer treatment. Int J Nanomed.

